# Gibson and Russell’s Physical Diagnosis

**Published:** 1903-06

**Authors:** 


Gibson and Russell's Physical Diagnosis.—Third edition revised and re-written. By Francis D. Boyd, C. M. G., M. D., F. R. C. P., Ed.; Assistant Physician, Edinburgh Royal Infirmary, etc. With 144 illustrations. Pp., 448. Price, $2.00. New York: D. Appleton & Co. 1902.

This book has been brought up to date in the present edition by adding several new sections: "Examination of the Blood," the "Examination of the Gastric Contents," "Intestinal Parasites," the "Cranial Nerves," and on "Clinical Bacteriology." The illustrations have also been increased to fit the new matter.

Looking over the volume, one finds nothing to criticise; on the contrary, he finds everything to commend. It is a condensed, practical little book. T. J. B.

